# TMB and BRAF mutation status are independent predictive factors in high-risk melanoma patients with adjuvant anti-PD-1 therapy

**DOI:** 10.1007/s00432-022-03939-w

**Published:** 2022-02-22

**Authors:** Julia Eckardt, Christopher Schroeder, Peter Martus, Sorin Armeanu-Ebinger, Olga Kelemen, Axel Gschwind, Irina Bonzheim, Thomas Eigentler, Teresa Amaral, Stephan Ossowski, Olaf Rieß, Lukas Flatz, Claus Garbe, Andrea Forschner

**Affiliations:** 1 Department of Dermatology, University Hospital of Tübingen, Liebermeisterstr. 25, 72076 Tübingen, Germany; 2 Institute for Clinical Epidemiology and Applied Biometrics, University Hospital Tübingen, Silcherstr. 5, 72076 Tübingen, Germany; 3 Institute of Medical Genetics and Applied Genomics, University Hospital Tübingen, Calwerstr. 7, 72076 Tübingen, Germany; 4 Institute of Pathology and Neuropathology, University Hospital Tübingen, Liebermeisterstr. 8, 72076 Tübingen, Germany; 5 Department of Dermatology, Charité Berlin, Luisenstr. 2, 10117 Berlin, Germany

**Keywords:** Anti-PD-1, Adjuvant, Melanoma, Checkpoint inhibition, Tumor mutational burden, RFS

## Abstract

**Background:**

High tumor mutational burden (TMB) is associated with a favorable outcome in metastatic melanoma patients treated with immune checkpoint inhibitors. However, data are limited in the adjuvant setting. As BRAF mutated patients have an alternative with targeted adjuvant therapy, it is important to identify predictive factors for relapse and recurrence-free survival (RFS) in patients receiving adjuvant anti-PD-1 antibodies.

**Methods:**

We evaluated 165 melanoma patients who started adjuvant anti-PD-1 antibody therapy at our center between March 2018 and September 2019. The initial tumor stage was assessed at the beginning of therapy according to the 8th edition of the AJCC Cancer Staging Manual. Tumor and normal tissue of the high-risk stages IIIC/D/IV were sequenced using a 700 gene NGS panel.

**Results:**

The tumor stages at the beginning of adjuvant anti-PD-1 therapy were as follows: *N* = 80 stage IIIA/B (48%), *N* = 85 stage IIIC/D/IV (52%). 72/165 patients (44%) suffered a relapse, 44/72 (61%) with only loco regional and 28/72 (39%) with distant metastases. Sequencing results were available from 83 to 85 patients with stage IIIC/D/IV. BRAF mutation status (HR 2.12, 95% CI 1.12–4.08; *p* = 0.022) and TMB (HR 7.11, 95% CI 2.19–23.11; *p* = 0.001) were significant and independent predictive factors for relapse-free survival (RFS).

**Conclusion:**

BRAF mutation status and TMB were independent predictive factors for RFS. Patients with BRAF V600E/K mutation and TMB high had the best outcome. A classification based on BRAF mutation status and TMB is proposed to predict RFS in melanoma patients with adjuvant anti-PD-1 therapy.

## Introduction

Immune checkpoint inhibitors (ICI) are effective therapies in stage III and IV melanoma patients with unresectable metastases. Nevertheless, lack of response is still a common problem as about 40–50% of the patients do not respond to combined ICI with ipilimumab and nivolumab (Wolchok et al. 2017; Robert et al. 2015; Ribas et al. 2016; Hodi et al. 2018).

Since the approval of anti-PD-1 therapies in the adjuvant setting, they are used frequently. However, there are also patients who suffer relapse despite adjuvant anti-PD-1 antibodies (Eggermont et al. 2018). The estimated 4-year recurrence-free survival (RFS) rate was 54% for BRAF mutated stage III patients receiving either adjuvant anti-PD-1 or adjuvant dabrafenib and trametinib therapy (Ascierto et al. 2020; Hauschild et al. 2018). Considering that for BRAF mutated stage III patients there is also the option of the BRAF and MEK inhibitors dabrafenib and trametinib in the adjuvant situation, the question arises if it might be possible to estimate the chance of benefit of adjuvant anti-PD-1 antibody therapy in advance (Long et al. 2017).

High tumor mutational burden (TMB) is associated with a favorable outcome in metastatic melanoma patients treated with immune checkpoint inhibitors, which could be due to a higher number of immunogenic antigens in TMB-high tumors (Snyder et al. 2014; Forschner et al. 2019; Samstein et al. 2019; Hugo et al. 2017). However, in targeted treated patients, the outcome was better at lower TMB values as demonstrated for example in the COMBI-AD study. In this study, patients with TMB-low had a prolonged relapse-free survival (RFS) with adjuvant dabrafenib and trametinib therapy, compared to patients with TMB-high tumors, what might be explainable by an increased tumor heterogeneity in TMB-high tumors with the consequence of more potential escape mechanisms (Dummer et al. 2020).

On the one hand, in patients with stage III and stage IV melanomas, the presence of BRAF mutation has been shown to be associated with a worse outcome compared to patients with BRAF wildtype melanomas (Hugdahl et al. 2016; Long et al. 2011; Ekedahl et al. 2013; Moreau et al. 2012). On the other hand, there is some evidence that ICI might be more effective in BRAF mutated melanomas compared to BRAF wildtype melanomas as the 5 year overall survival rate was 60% for BRAF mutant melanoma patients with combined ipilimumab and nivolumab therapy compared to 48% in BRAF wildtype patients as shown in the CheckMate 067 study (Hodi et al. 2018).

However, data are limited in the adjuvant setting. As BRAF mutated patients have an alternative with adjuvant targeted therapy, predictive factors for relapse and RFS in patients receiving adjuvant anti-PD-1 antibodies would be helpful. In this study, we sought to explore the relevance of TMB and BRAF mutation status in terms of relapse and RFS in patients receiving adjuvant anti-PD-1 antibody therapy.

## Materials and methods

The study was approved by the local Ethics Committee of the University of Tübingen (project number 606/2020BO).

### Data collection

All melanoma patients (*n* = 165) who started adjuvant anti-PD-1 antibody therapy at our center of dermato-oncology between 1st of March 2018 and 30th of September 2019 were included in this study. The patients were cared for and treated according to the German melanoma guidelines recommending complete lymph node dissection (CLND) for patients with sentinel lymph node metastases > 1 mm or clinically detected lymph node macrometastasis (Leitlinienprogramm Onkologie, Deutsche Krebsgesellschaft, Deutsche Krebshilfe, AWMF): Diagnostik, Therapie und Nachsorge des Melanoms, 2020). Three patients with sentinel lymph node metastases > 1 mm refused CLND and one patient underwent CLND despite sentinel node metastasis ≤ 1 mm. Adjuvant radiotherapy after CLND was offered as recommended in the German melanoma guidelines in case of (i) three or more metastatic lymph nodes, (ii) diameter of metastasis > 3 cm or (iii) extracapsular spread of tumor cells (Samstein et al. 2019; Garbe, et al. 2016; Gershenwald and Scolyer 2018). Patients with manifest tumor cells only detected by quantitative immune-cytology were classified stage IIIA (Ulmer 2014).

The clinical data collection on sex, age, type of melanoma, date of diagnosis, previous and further therapies, stage at start of adjuvant anti-PD-1 therapy, type of ant-PD-1 antibody and site, type and date of relapse was carried out by evaluation of the electronic patient database by clinical experienced dermato-oncologists (JE, AF).

Since NGS of sufficient quality is more difficult in stage IIIA and B patients due to the usually very limited amount of tumor material available in the form of micrometastases, we focused on the high-risk stages IIIC/D and IV and decided to perform NGS in all patients of this subgroup.

### Next-generation-sequencing

Next generation sequencing (NGS) of the tumor and blood of all stage IIIC/D/IV patients was performed in the Institute of Medical Genetics and Applied Genomics Tübingen. We focused on stage IIIC/D/IV because we were able to arrange NGS for all patients in this subgroup. Stage IIIA and B include predominantly patients with low tumor burden in the lymph node and a lower risk of recurrence, therefore NGS is not routinely performed in the context of clinical care. In two patients of the stage IIIC/D/IV NGS analysis was not possible (1 × insufficient tumor content, 1 × lack of patient consent). DNA was isolated from tumor FFPE material and blood following standard protocols: Genomic DNA was extracted from macrodissected 5 µm paraffin sections using the Maxwell^®^ RSC DNA FFPE Kit and the Maxwell^®^ RSC Instrument (Promega, Madison, WI, USA) according to the manufacturer’s instructions. 200 ng of genomic DNA (gDNA) were fragmented with a Covaris Ultrasonicator instrument (Covaris, Woburn, MA, USA). Regions of interest were enriched using the SureSelect XT Low Input Target Enrichment System (Agilent Technologies, Santa Clara, CA, USA) covering 708 cancer related genes, 7 promoter regions and selected fusions. Sequencing data was analyzed using the in-house meg-SAP Pipeline (https://github.com/imgag/megSAP, https://github.com/imgag/ngs-bits). In summary, sequencing reads were aligned to the human reference genome (GRCh37) by BWA-MEM (Li and Durbin 2009). Variants were called using Strelka2 (Kim et al. 2018) and annotated with VEP (McLaren et al. 2016). ClinCNV (Demidov and Ossowski 2019) was used for detection of somatic copy number variants. Tumor mutational burden was calculated as previously described (Forschner, et al. 2020). According to Samstein et al., TMB “high” was defined as the top 20% of the cohort (Samstein et al. 2019), corresponding to TMB values of ≥ 20 Var/Mb in this cohort.

### Statistical analysis

Statistical analysis was performed by using SPSS version 25 (SPSS Inc., Chicago, IL, USA). TMB and BRAF mutation status were tested as predictive factors for RFS by Cox regression analysis. RFS time was defined as the time between first cycle of anti-PD-1 antibody and relapse, melanoma specific death, or censored at the last date of patient contact. The occurrence of a relapse was counted as event. All relapses occurred before any melanoma-specific death. We built combined variables (TMB-high + BRAF mutation, TMB-high + BRAF wildtype, TMB-low + BRAF mutation, TMB-low + BRAF wildtype) and estimated RFS according to the Kaplan–Meier method. Differences between groups were tested using the Log rank test for trend. The level of significance was 0.05 (two-sided) in all analyses.

## Results

### Patient cohort

165 patients with Stage IIIA-IV melanoma receiving adjuvant PD-1 antibody therapy between March 2018 and September 2019 in our department were included in this retrospective single centre study. Patients’ characteristics are summarized in Table 1. The median age at primary diagnosis was 60 years (range 17–89 years) and about half of the patients (45%) were female. The most frequent melanoma subtype was cutaneous melanoma (*n* = 127, 77%), 24 patients (15%) had occult melanoma with unknown primary, 9 patients (5%) acral melanoma and 5 patients (3%) mucosal melanoma.

**Table 1:** Patients’ characteristics of 165 stage IIIA-D/IV melanoma patients with adjuvant anti-PD-1 therapy

Characteristic	*N*	%
Total cohort	165	100
Sex		
Female	75	45
Male	90	55
Melanoma subtype		
Cutaneous	127	77
Occult	24	15
Acral	9	5
Mucosal	5	3
Stage at start of adjuvant PD-1 antibody		
Stage IIIA	19	12
Stage IIIB	61	37
Stage IIIC	70	42
Stage IIID	5	3
Stage IV	10	6
Therapy-related adverse event		
No	119	72
Yes	46	28
Regional lymphadenectomy		
No	93	56
Yes	72	44
Adjuvant radiotherapy		
No	130	79
Yes	35	21
Type of adjuvant PD-1 antibody		
Nivolumab	155	94
Pembrolizumab	10	6

27 patients (16%) had received low dose interferon-α as prior adjuvant treatment. 72 patients (44%) underwent complete lymph node dissection (CLND) because of lymph node metastasis and 35 patients (21%) received additional adjuvant radiotherapy. Most of the patients (94%) received nivolumab as adjuvant anti-PD-1 antibody. The median time between primary diagnosis and start of the adjuvant therapy was 7 months (range 1–407 months). Most of the patients (*n* = 119, 72%) had no immune-related adverse events. Adjuvant PD-1 antibody therapy had to be stopped in 12 patients (7%) due to severe therapy-related adverse events. The median follow-up since start of adjuvant PD-1 antibody therapy was 22 months (range 7–23 months).

**Fig. 1.**
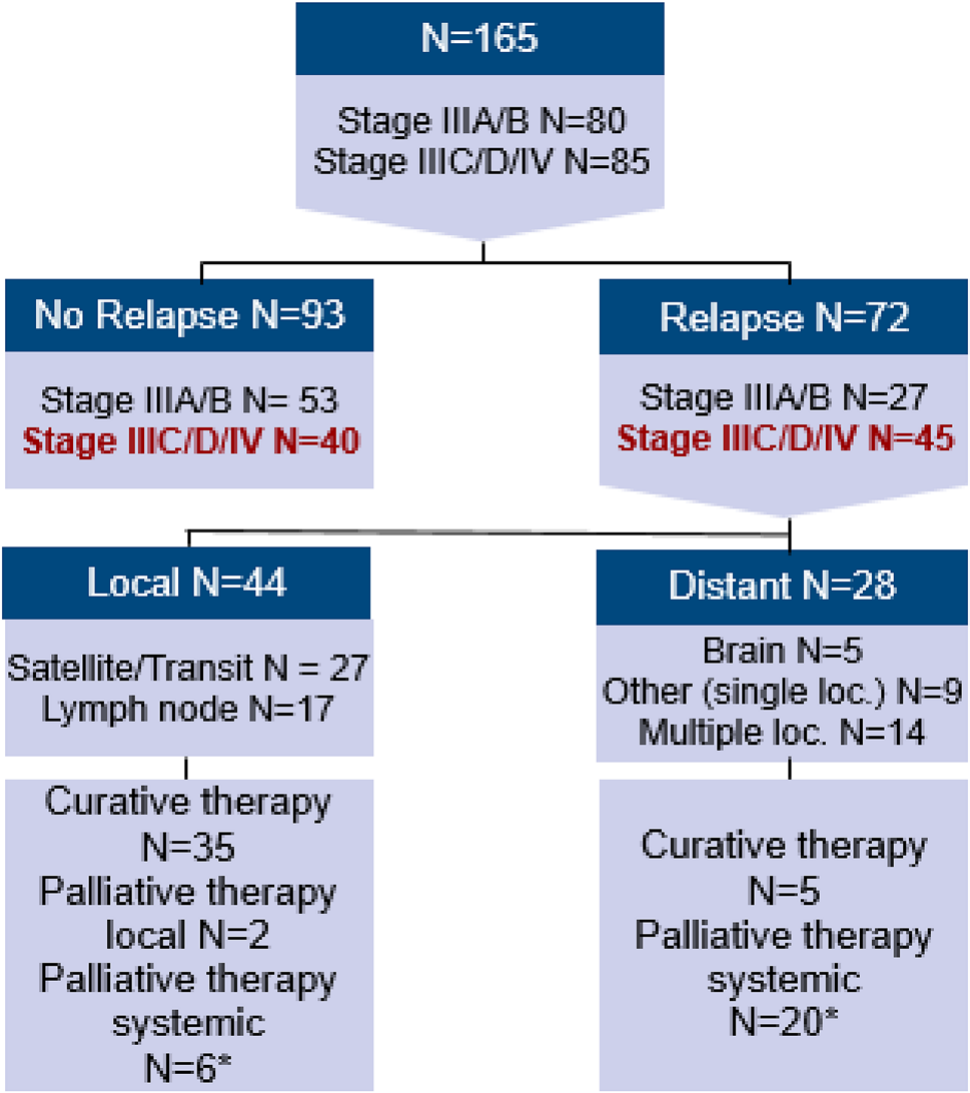
Stage at start of adjuvant anti-PD-1 therapy, pattern of relapse and further therapies. *further therapies: lost for follow-up in 4/72 patients

72 patients (44%) suffered from relapse under anti-PD-1 antibody therapy. In the stage IIIA/B melanoma group 27 patients (34%) had a relapse and in the stage IIIC/D/IV group 45 patients (53%). The localization of relapse was as follows: 44/165 patients (27%) had only locoregional relapse (regional lymph node, satellite or transit metastases) and 28/165 patients (17%) suffered distant metastases with/without additional locoregional relapse. 59 patients (36%) suffered relapse during the 12 months of adjuvant anti-PD-1 antibody therapy, 11 patients (7%) after completion of 12 months adjuvant therapy and two patients (1%) suffered relapse after having discontinued adjuvant treatment due to adverse events. Patients’ stages at start of adjuvant anti-PD-1 antibody therapy, pattern of relapse and further therapies in patients with relapse are displayed in Fig. 1.

In most of the patients with locoregional relapse, curative therapy was possible. Surgery of metastases was performed in 35 of 44 patients (80%), 26 patients of them received afterwards another course of adjuvant anti-PD1 therapy. In four patients with BRAF mutation, adjuvant targeted therapy was initiated instead of a second course of anti-PD-1 antibody therapy. 6 of 44 patients (14%) received non-adjuvant ICI or targeted therapy (TT).

Among the patients with distant metastasis, 5 of 28 patients (18%) received curative surgery followed by another course of adjuvant anti-PD-1 antibody therapy. However, most of the patients had unresectable metastases and received palliative combined ICI with Ipilimumab and nivolumab or TT. One patient was referred to chemotherapy (Supplemental Tab. 1).

## Stage IIIC/D/IV cohort

All stage IIIC/D/IV patients (*n* = 85) were referred to Next generation sequencing (NGS). Clinical characteristics, TMB and BRAF mutation status are displayed in Table 2.

**Table 2:** Characteristics of stage IIIC/D/IV cohort

Characteristic	*N*	%
Total cohort	85	100
Sex		
Female	36	42
Male	49	58
Melanoma subtype		
Cutaneous	63	74
Occult	12	14
Acral	6	7
Mucosal	4	6
BRAF mutation status		
BRAF V600 E/K mutation present	32	38
BRAF V600 E/K mutation absent	51	60
Missing	2	2
Tumor mutational burden (TMB)		
TMB high (≥ 20 Var/Mb)	19	22
TMB low (< 20 Var/Mb)	64	75
Missing	2	2
	Median	IQR
Age at first diagnosis (years)	64	51–73
Follow-up since start of anti-PD-1 antibody (months)	22	17–26
Relapse-free survival (months)	15	5–23
Time to relapse (months)	5	3–10
Tumor mutational burden (TMB; Var/MB)	10	5–18

## TMB and BRAF mutation status are predictive factors for relapse-free survival

**Fig. 2.**
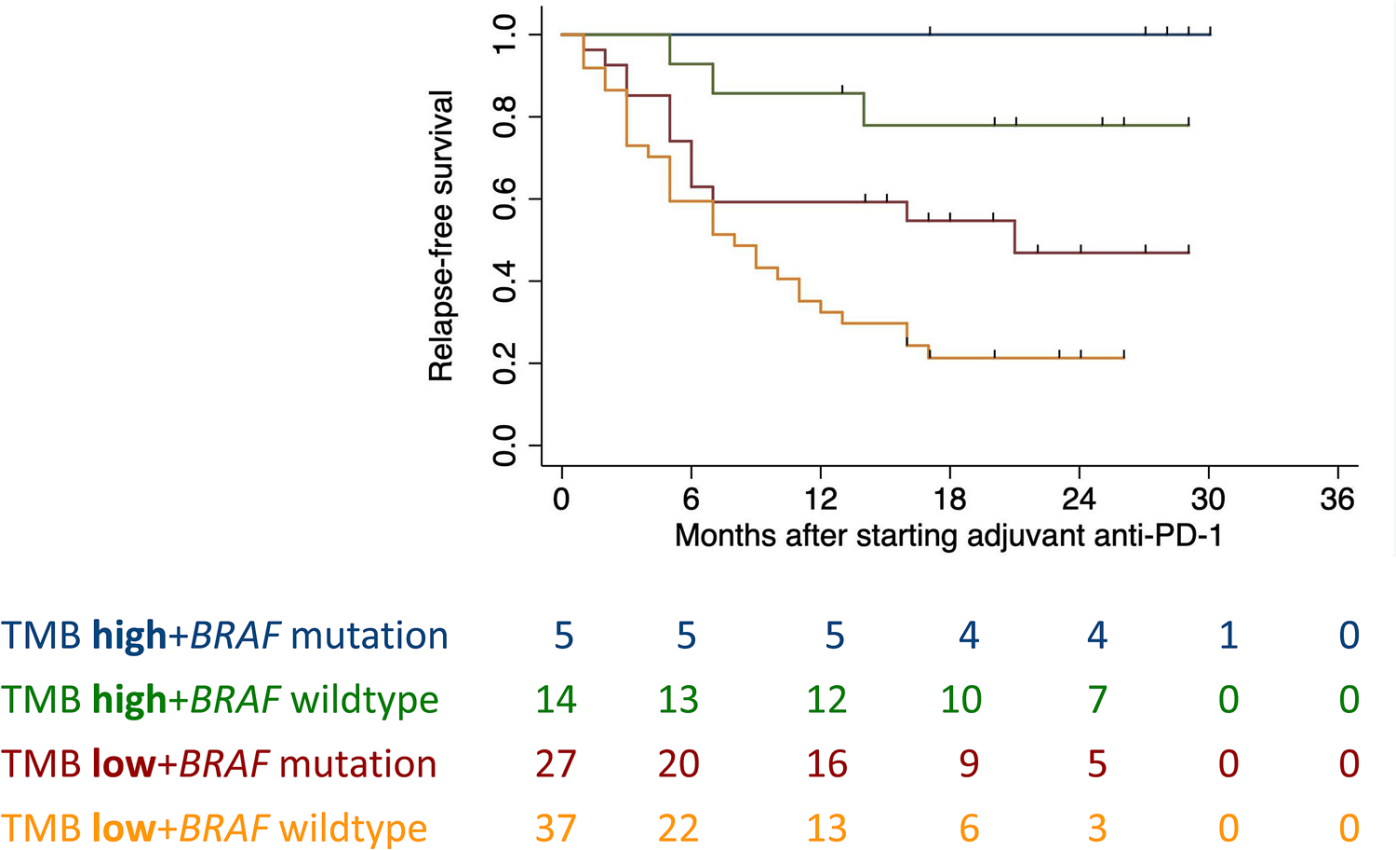
Impact of Tumor mutational burden (TMB) and BRAF mutation status on relapse-free survival of the stage IIIC/D/IV patients

With regards to RFS, BRAF mutation status (HR 2.12, 95% CI 1.12–4.08; *p* = 0.022) and TMB (HR 7.11, 95% CI 2.19–23.11; *p* = 0.001) were statistically significant and independent predictive factors (Fig. 2). The table below displays the results for combined variables and RFS (Tab. 3). Patients with BRAF V600E/K mutation and TMB-high had the best outcome.

**Table 3:** Tumor mutation burden (TMB), BRAF status and 1- and 2-year relapse-free survival of the stage IIIC/D/IV patients

Stage IIIC/D/IV	1 year RF S% (95% CI)	2-year RFS % (95% CI)	*P*
TMB-high + BRAF mutation	100	100	< 0.0001
TMB-high + BRAF wildtype	86 (67–100)	78 (56–100)	
TMB-low + BRAF mutation	59 (41–78)	47 (25–69)	
TMB-low + BRAF wildtype	32 (15–48)	21 (8–35)	

The 1- and 2-year RFS was 100% for the patients with TMB-high and BRAF mutated tumors. In contrast, RFS was significantly worse for patients with TMB-high and BRAF wild type and even more for those with TMB-low and BRAF wild type melanomas.

## Discussion

We found that TMB and BRAF mutation status are independent predictive factors for RFS of stage III/IV melanoma patients with adjuvant PD-1 antibody therapy.

Both, ICI and TT with Dabrafenib and Trametinib are approved in the adjuvant setting for melanoma patients with stage III melanoma after resection of lymph node metastases. Furthermore, Nivolumab is approved for stage IV melanoma patients in the adjuvant setting (Eggermont et al. 2018; Ascierto et al. 2020;  Long et al. 2017).

Compared to the EORTC 1325/KEYNOTE-054 study, that included stage III melanoma patients with adjuvant pembrolizumab after CLND, we observed a similar recurrence rate (RR) but more locoregional and less distant metastases. In our study 44% of the patients suffered relapse (27% locoregional and 17% distant), in the Keynote-054 study 37% of the patients suffered relapse, 13% locoregional and 23% distant. This difference might be due to a lower rate of CLND in our study compared to 100% in the Keynote-054 study. Furthermore, median follow-up in the Keynote-054 study was 36.6 months, thus considerably longer than in our study (median follow-up 22 months) (Eggermont et al. 2020). In the Checkmate238 study with completely resected stage IIIB/C and IV melanoma patients receiving adjuvant nivolumab or adjuvant ipilimumab, 47% recurrences were observed in the nivolumab group and 56% in the ipilimumab group with distant metastases as the most common type of recurrence in both groups (Ascierto et al. 2020). Therefore, the recurrence rates (RR) of the comparable studies correspond very well to that of our study (RR 44%), considering that Keynote-054 (RR 37%) included only stage III, Checkmate 238 (RR 47%) stage IIIB/C and IV melanoma patients. Nevertheless, in both of the studies there were more distant relapses and less local relapses compared to our study.

Treatment-related adverse events can be challenging and ICI might have to be interrupted or stopped due to immune-related adverse events. In our study, 28% of the patients developed immune-related adverse events and in 7% of the patients, adjuvant anti-PD-1 therapy had to be stopped because of immune-related adverse events. These results correspond very well to the occurrence of grade 3–4 adverse events in 27% of the adjuvant nivolumab group of the IMMUNED study. However, a remarkably high percentage of 71% of grade 3 or 4 adverse events was observed in the combination arm with adjuvant ipilimumab and nivolumab of this study, thus considerably higher compared to combined ipilimumab and nivolumab for unresectable metastases (Zimmer et al. 2020). The discontinuation rate of adjuvant nivolumab due to adverse events was 7.7% in the Checkmate 238 study, which is similar to our results (Weber et al. 2017).

BRAF V600 mutations are present in about 50% (43–66%) of primary or metastatic melanoma (Long et al. 2011; Colombino et al. 2012; Davies et al. 2002), thus the rate of 42% BRAF V600 mutated tumors in our study corresponds very well to the literature. The prognostic role of BRAF mutations is controversial. Some studies report a more aggressive clinical course in BRAF mutated melanoma and a worse outcome compared to BRAF-wildtype melanoma (Long et al. 2011; Ekedahl et al. 2013; Barbour et al. 2014). This was also confirmed for stage III melanoma patients. In the study of Babour et al., BRAF mutated patients had a significant higher recurrence rate (77%) after three years of follow-up, than BRAF wild-type patients (54%; hazard ratio 1.8, *p* = 0.008) after CLND in a cohort of 124 stage IIIB/C melanoma patients. 3 year distant recurrence rate and local nodal recurrence rates were 73 and 20% for the BRAF mutant group versus 42% and 8% for the BRAF wild-type group (*p* < 0.001, *p* = 0.15) (Barbour et al. 2014). Picard and colleagues also showed a significant difference in overall survival between the BRAF-mutated and wild-type patients in a study with 72 melanoma patients after positive sentinel node dissection (Picard et al. 2014). Other studies report no association of BRAF mutation status and prognosis (Meckbach 2014; Carlino et al. 2014).

Concerning the predictive value of the BRAF mutation status in patients with adjuvant ICI, in the Checkmate-238 study, RFS was similar in nivolumab-treated BRAF mutated or BRAF wildtype patients (Weber et al. 2017). However, in the CheckMate 067 study it was demonstrated that the 5 year overall survival rate was 60% for BRAF mutant melanoma patients with combined ipilimumab and nivolumab therapy compared to 48% in BRAF wildtype patients, indicating a positive predictive value for BRAF mutant melanoma (Hodi et al. 2018). In the IMMUNED study BRAF mutated patients had a better outcome for combined ipilimumab and nivolumab therapy compared to nivolumab (Zimmer et al. 2020). A positive predictive value for BRAF mutant melanoma with ICI corresponds well to our data indicating that BRAF mutant melanoma and even more if additional TMB-high, had the best RFS.

TMB-high melanoma is associated with a higher overall response rate to ICI with anti- PD-1 and/or anti-CTLA-4 antibodies and improved overall survival. The positive predictive value of TMB-high in ICI treated patients was also shown in other tumors than melanoma compared to a low TMB. A high TMB is thought to lead to a higher number of immunogenic antigens, thus improving immune-related responses on PD-1 and/or CTLA-4 antibodies (Snyder et al. 2014; Forschner et al. 2019; Samstein et al. 2019; Hellmann et al. 2018). Therefore, the results of our study, indicating a positive predictive value and improved RFS for adjuvant anti-PD-1 treated patients whose tumors are TMB-high, match to the results in the literature.

In contrast, the data on adjuvant dabrafenib and trametinib in the COMBI-AD study showed improved RFS in stage III melanoma with TMB-low tumors, presumably due to less tumor heterogeneity (HR [versus placebo] 0·49, 95% CI 0·35–0·68, *p* < 0·0001) (Dummer et al. 2020).

Similarly, it has been observed in other tumors with targeted therapies that the outcome was better for tumors with a lower TMB, such as EGFR-mutated metastatic lung cancer with EGFR tyrosine kinase inhibitors: Offin et al. demonstrated that the TMB value was inversely associated with the clinical outcome in these patients treated with EGFR tyrosine kinase inhibitors (Offin et al. 2019).

BRAF mutated melanomas with a high tumor mutation burden have a great chance to benefit from adjuvant anti-PD-1 therapy. In contrast, if the TMB value is low, such patients might be more likely to benefit from adjuvant BRAF and MEK inhibitors, assuming that their tumor tissue is less heterogeneous. However, there are no head-to-head studies on this topic and therefore, in BRAF mutated patients both adjuvant treatment options have to be discussed with the patients.

The strength of our study consists in a complete and comprehensive survey of all patients treated with anti-PD-1 antibodies at our centre in a defined follow-up time of 22 months and only very few (*n* = 4) patients that were lost for follow-up. Therefore, the risk of bias concerning for example patients with excellent or fatal courses can be excluded. All patients with stage IIIA-D or IV at the beginning of adjuvant PD-1 antibody therapy were included in this real-world data set. Second, calculation of TMB by a 700 gene panel with a target size of > 3 Mb is very reliable, compared to panels of smaller size (Buchhalter et al. 2019).

This study has also some limitations. Only therapy-related adverse events requesting therapeutic consequences such as corticosteroid therapy or treatment interruptions were documented in the patients’ electronic files. Mild adverse events without consequences might be missing. Furthermore, CTCAE grading could not be completely captured due to the retrospective study design.

The study consists of a single centre cohort with a limited number of patients. It would be desirable if our results could have been validated in an independent multicentre cohort. Currently there is no such option due to lack of studies, but such data will certainly be available in the near future, for example by evaluating the large cohort of TRIM-ADJUVANT (Ugurel and Mohr 2020).

## Conclusion

In this study, TMB and BRAF mutation status were identified as independent, predictive factors for relapse-free survival in high-risk melanoma patients with adjuvant anti-PD-1 therapy. Both factors can be combined leading to a classification of four groups whose 2-year relapse-free survival rates range from 100 to 21%. Therefore, TMB and BRAF mutation status can help in the selection of adjuvant therapies and surveillance regimen.

## Data Availability

The datasets used and/or analyzed during the current study are available from the corresponding author on reasonable request.
